# Functional Mobility Studies in Younger Adults: Instrumented Timed Up and Go (iTUG) Test Using Inertial Devices

**DOI:** 10.3390/jcm14061944

**Published:** 2025-03-13

**Authors:** Mateusz Kowal, Sławomir Winiarski, Ewa Morgiel, Marta Madej, Krzysztof Proc, Marcin Madziarski, Nicole Wedel, Agata Sebastian

**Affiliations:** 1 Physiotherapy Research Laboratory, University Centre of Physiotherapy and Rehabilitation, Faculty of Physiotherapy, Wroclaw Medical University, 50-368 Wroclaw, Poland; mateusz.kowal@umw.edu.pl; 2 Department of Physiology and Biomechanics, Wroclaw University of Health and Sport Sciences, Paderewskiego 35, 51-612 Wrocław, Poland; 3 Department and Clinic of Rheumatology and Internal Medicine, Wroclaw Medical University, Borowska Street 213, 50-556 Wroclaw, Poland; ewa.morgiel@umw.edu.pl (E.M.); marta.madej@umw.edu.pl (M.M.); agata.sebastian@umw.edu.pl (A.S.); 4 Department and Clinic of Rheumatology and Internal Medicine, University Hospital, Borowska Street 213, 50-556 Wroclaw, Poland; 5 Department of Medicine, Albert Einstein College of Medicine, 1300 Morris Park Ave, New York, NY 10461, USA; nicole.wedel@einsteinmed.edu

**Keywords:** motion analysis, functional mobility assessment, normative data

## Abstract

Functional Mobility Assessment (FMA) is a challenging task. One example of an FMA is an instrumented Timed Up and Go test (iTUG). Sensor-based interventions are more effective than traditional interventions that use clinical tests to assess a patient’s FMA. **Background/Objectives**: The aim of this study is to investigate the variability of selected parameters of the instrumented Time Up and Go test using inertial measurements in healthy younger adults. **Methods**: A total of 73 subjects participated in the study, including 37 women and 36 men. The mean age was 31 years (SD 5.5 years), the mean height [cm] was 176.2 (SD 91), and the mean BMI [kg/m^2^] was 26.6 (SD 3.1). The Noraxon MyoMotion Research 18 motion analysis system was used to record raw spatial data. **Results**: The mean total time to complete the iTUG test was 13.1 ± 1.9 s with a low coefficient of variation (CV), suggesting consistent performance between participants. The recorded spatial and temporal parameters of the gait variables, as well as the kinematic variables of the iTUG test of the studied group of healthy adults, show low variability, except for the mean double support ratio (R − L)/(R + L), which was 4.1 ± 11.0% with a CV of 271.5%, indicating a very high variability. **Conclusions**: The low variability observed in key parameters, such as total time and percentage of posture, suggests that the iTUG test provides reliable, objective, and reproducible measurements that can serve as normative benchmarks for healthy adults.

## 1. Introduction

Assessing functional mobility is a challenging task. The Functional Mobility Assessment (FMA) measures the skills, behaviours, and knowledge required to achieve self-sufficiency and meet basic needs. This term encompasses various tests and measures that evaluate an individual’s ability to perform tasks necessary for daily living, focusing on balance, gait, and overall physical function. Functional mobility outcomes are often treated as a criterion for evaluating the effectiveness of therapeutic interventions. Therefore, tests designed to assess FMA must be simple to perform and interpret. Several quantitative measures of gait and balance have been introduced to determine FMA, including normal gait speed, the Berg Balance Scale (BBS), the Six-Minute Walk Test (6MWT), the Falling Efficacy Scale-International (FES-I), the Tinnetti Test, and the Timed Up and Go (TUG) test [1,2]. The TUG test is one of the most commonly used tests for assessing FMA [3,4]. The TUG test is designed to measure several critical components of FMA. For one, it assesses an individual’s ability to walk a short distance, providing valuable insights into overall mobility and gait efficiency. Additionally, the test evaluates dynamic balance, particularly during transitions such as sitting to standing, walking, turning, and returning to a seated position. These transitions are crucial in daily life, and the ability to perform them independently offers an important measure of functional independence. Furthermore, the duration taken to complete the TUG test is often correlated with fall risk; longer completion times typically indicate a higher risk of falls [4]. This makes the TUG test a valuable tool for identifying individuals who may benefit from targeted fall prevention interventions. The clinical utility and widespread popularity of the TUG test are supported by extensive research across various fields, including geriatrics [5,6], neurology [7,8,9], orthopaedics and rheumatology [10,11,12], and general surgery [13]. Its popularity stems from its ability to easily assess mobility and balance in patients with various conditions.

Using sensor-based measurement tools is statistically more effective than traditional measurement using clinical tests to assess the patient [14]. An example is the instrumented Timed Up and Go (iTUG), during which sensors are used in a typical TUG test. Sensor-based technologies can be divided into three types: optical sensors, perception sensors, and wearable sensors (e.g., Inertial Measuring Unit, IMU). There is a wide range of applications for iTUGs in clinical practice, including assessing fall risk and therapy progress [14,15,16,17,18,19,20]. Current evidence suggests that therapy using sensor-based technologies improves outcomes for people who have received biofeedback [21,22,23]. Therefore, it may be useful to obtain normative data from healthy adults to distinguish abnormal from normal results. So far, IMU and optical sensor devices have been used mainly in iTUG assessment. In a systematic review and meta-analysis, Kobsar et al. identified areas that they believe should be developed to increase knowledge about the use of IMU in FMA research [24]. The authors suggest that future research should focus less on providing information on validity and reliability and more on publishing the work results on protocols, as joint kinematics generally demonstrate good to excellent validity and reliability.

Unlike previous investigations focusing on healthy elderly individuals, this study emphasises normative values derived from a younger adult population, addressing a gap identified in recent systematic reviews [25]. This study investigates the variability of selected parameters of the instrumented Time Up and Go test using inertial measurements in healthy younger adults. In addition, we want to answer the following questions: What are these systems’ roles, and what diagnostic information can they provide? The information on normative data will be useful to future researchers and those performing the iTUG test in their daily clinical work. A significant advancement in clinical practice and research has been achieved with the integration of wearable inertial measurement units (IMUs), which could provide more detailed and precise measurements of the TUG test’s various parameters, enhancing its applicability and accuracy in both settings [26,27,28,29,30,31].

## 2. Materials and Methods

### 2.1. Setting and Ethical Considerations

All tests were performed in 2021–2023 in a certified Biomechanical Analyses Laboratory of the Department of Biomechanics at the Academy of Physical Education in Wroclaw (Poland) under controlled environmental conditions. The laboratory maintained a temperature of approximately 22 °C with a relative humidity of 50%. The test area had a flat, unobstructed surface with uniform lighting to minimise potential external influences on movement performance. The study followed the Declaration of Helsinki’s ethics guidelines and principles and was approved by the Bioethics Committee at the Medical University of Wroclaw (approval number KB-1080/2021). All study participants were informed of the purpose of the study and the approach to be used, and all participants signed an informed consent form.

### 2.2. Participants

The final studied sample included 73 participants (37 women and 36 men) with a mean age, body mass, body height, and BMI of 31 ± 5.5 years, 68.4 ± 4.3 kg, 176.2 ± 9.1 cm, and 26.6 ± 3.1 kg/m^2^, respectively.

The inclusion criteria were that the participants had to be between 20 and 40 years of age and be physically active with 150 to 300 min of moderate-intensity aerobic physical activity. The exclusion criteria were a history of musculoskeletal, nervous system, or other diseases affecting functional ability, or the presence of a cognitive disorder preventing understanding of the task to be performed or obtaining informed consent. Subjects with a BMI over 31 were also excluded from the study.

### 2.3. Procedure

All measurements were conducted under standardised laboratory conditions. Before the test, participants were briefed on the procedures and allowed to ask questions. A detailed explanation of the test was given, including a demonstration of the movements required. The sequence of steps was as follows:

- Participants were seated in a standard chair with armrests, with their feet resting flat on the floor.
- They were instructed to stand up upon a verbal command (‘Go’), walk a distance of 3 m (measured and marked on the floor), turn around at the designated marker, and return to the chair to sit down.
- Measurements were taken one participant at a time to minimise distractions and ensure accurate motion capture.
- Participants performed the test at their self-selected normal walking speed. They were instructed not to run but to perform the task as quickly and safely as possible.

The test distance of 3 m was based on standardised TUG protocols commonly used in Europe, aligning with existing clinical guidelines. Notably, differences in TUG protocols between Europe (3 m) and the USA (10 feet, approximately 3.048 m) are minimal and do not significantly impact test outcomes.

### 2.4. Data Analysis

**Figure 1:**
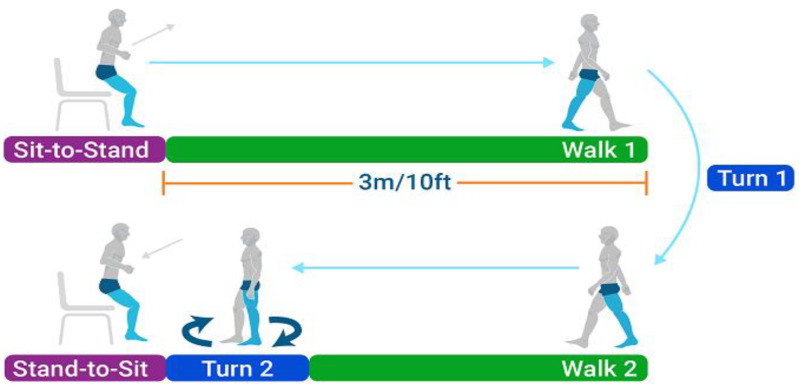
Schematic representation of the Instrumented Timed Up and Go (iTUG) test phases. The test consists of several distinct phases: (1) Sit-to-Stand: the participant rises from a seated position, (2) Walk 1: the participant walks a distance of 3 m/10 feet, (3) Turn 1: the participant turns 180 degrees, (4) Walk 2: the participant walks back the same distance, (5) Turn 2: the participant turns 180 degrees to face the chair, and (6) Stand-to-Sit: the participant returns to a seated position.

The test begins with the subject seated on a chair, transitioning into the first phase, termed the “Sit-to-Stand” phase (Figure 1). This phase involves rising from a seated position, requiring an assessment of lower limb strength and balance control. Following the “Sit-to-Stand” phase, the subject enters the “Walk 1” phase, which entails walking a distance of circa 3 m in a straight line. This phase is crucial for evaluating gait parameters. Upon reaching the 3-metre mark, the subject performs “Turn 1”, involving a 180-degree turn. This phase examines the subject’s balance and coordination. The next phase, “Walk 2”, involves walking back to the chair over the same 3-metre distance. As the subject approaches the chair, “Turn 2” is executed, another 180-degree turn preparing the subject for the final phase. Turn 2 also assesses balance and coordination, similar to “Turn 1”. The final phase, “Stand-to-Sit”, involves the subject returning to a seated position.

During the TUG test, kinematic parameters, including joint angles, segmental velocities, and spatial–temporal gait characteristics, are measured. The time schematic provides a detailed breakdown of the total time taken for each phase of the TUG test. The total time is segmented into the specific activities: Stand (cc 1.2 s, 9%), Walk 1 (cc 4.0 s, 32%), Turn 1 (cc 2.0 s, 16%), Walk 2 (cc 3.1 s, 25%), Turn 2 (cc 0.8 s, 7%), and Sit (cc 1.4 s, 11%), culminating in a total time of cc 12.5 s. This temporal distribution allows for a thorough analysis of each movement phase, aiding in assessing the subject’s overall mobility and functional performance.

### 2.5. Motion Analysis

The instrumented Timed Up and Go (iTUG) test was performed using the Myomotion 3D analysis system from Noraxon (Scottsdale, AZ, USA), which included 18 wireless IMUs capable of capturing three-dimensional motion data at a sampling rate of 200 Hz. The system was paired with myoRESEARCH software (version MR4), facilitating real-time data acquisition, processing, and synchronisation across all sensors.

A static calibration was conducted prior to each testing session. Participants stood in a standardised upright posture while the system established baseline positions for all sensors. Calibration data were used to correct for individual variations in limb length and joint orientation.

The iTUG protocol was programmed into the MR4 software to standardise data collection. This protocol included event markers for the ‘start’ (standing up), ‘mid-walk’ (completion of the 3 m walk), ‘turn’ (180-degree pivot), and ‘end’ (return to sitting).

Data were collected in a controlled indoor laboratory setting with a flat, unobstructed walking path marked for 3 m. Participants were instructed to perform the test at a comfortable walking speed, as described in the Procedures section.

**Figure 2:**
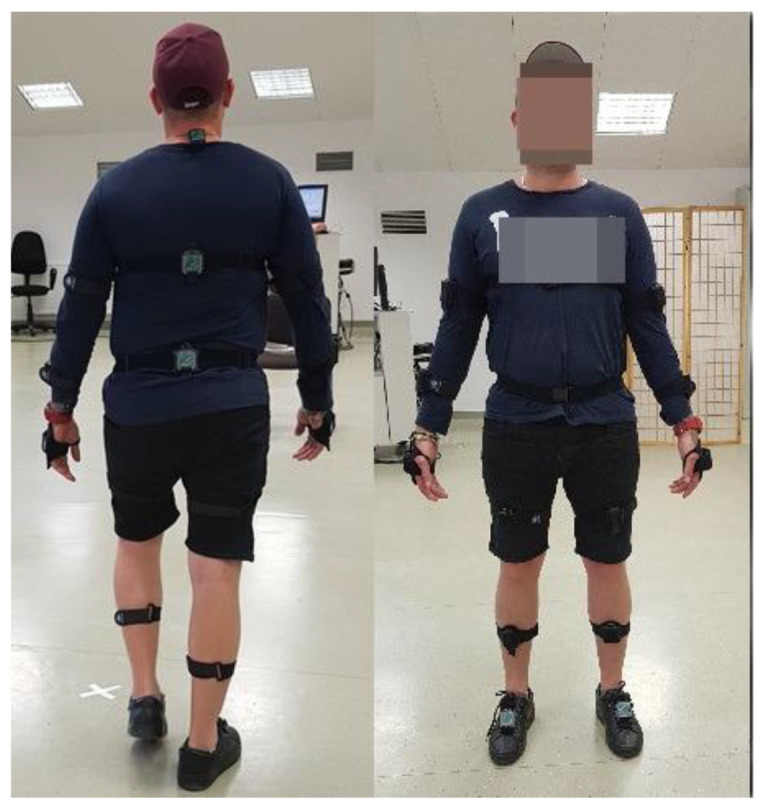
Illustrative placement of IMU sensors in front and rear views.

Data quality was monitored during each trial using the real-time visualisation feature in MR4 software. If artefacts or inconsistencies were observed, the trial was repeated after verifying sensor placement and calibration. Proper consideration of error and tolerance is important in order to ensure the most accurate and informative results [27]. The position of the sensors is shown in Figure 2 and Table 1.

The parameters characterising the Timed Up and Go (TUG) test are detailed in Table 2, which provides a comprehensive overview of the specific metrics assessed in each test phase. The table lists each parameter and its unit of measurement, as well as precise definitions and interpretations relevant to clinical practice. During data analysis and interpretation, these parameters can allow for quantitative assessment of movement efficiency, balance, and trunk stability throughout different phases of the TUG test. This structure facilitates comparisons across participants, highlighting deviations in standard movement patterns that may indicate functional limitations.

### 2.6. Statistical Calculations

Registered data curation involved filtering and organising the raw data from the Timed Up and Go (TUG) test to ensure consistency and accuracy prior to analysis. Following this, several key measures were calculated to summarise the dataset, including the mean value, which represents the average value of the dataset, providing a central tendency of the data. The median value was also calculated as the middle value when the data were ordered to represent the central point and help to identify skewness. In addition, Minimum and Maximum values defined the lower and upper bounds of the observed range, respectively, giving insight into the extent of observed values; the lower quartile Q1 representing the 25th percentile and the upper quartile Q3 at the 75th percentile provided insight into the range of values within the dataset particularly in the middle 50 percent. Finally, the quartile range or interquartile range was calculated as the difference between the upper and lower quartiles, which showed the spread of the central portion of the data. Additionally, the standard deviation (SD) measured the extent to which the data varied from the mean, giving a sense of the data’s dispersion around the mean. The coefficient of variation, calculated as the ratio of the standard deviation to the mean, was useful for comparing variability across datasets with different scales or units. Together, these measures provided a comprehensive understanding of the data’s distribution central tendencies and variability essential for interpreting performance trends and identifying potential outliers in the TUG test parameters

Descriptive statistical analyses were performed for key parameters of the instrumented Timed Up and Go (iTUG) test. These included measures of central tendency (median) and variability, specifically the lower quartile (Q1), upper quartile (Q3), interquartile range (IQR), and coefficient of variation (CV). These metrics were chosen to provide robust insights into the distribution and variability of the data, minimising the influence of potential outliers.

Sample size estimation was performed post hoc based on a pilot study of the Timed Up and Go (TUG) test conducted on 5 participants. Using a known mean and standard deviation of 12.1 ± 1.2 s, we applied the following formula to determine the required sample size for estimating a population mean with a 95% confidence level (Z = 1.96) and a margin of error (E) of ±0.3 s:

(1) $$Sample\;Size={\left(\frac{Z\cdot SD}{E}\right)}^{2}$$

Thus, a minimum of 62 participants was established.

## 3. Results

### 3.1. iTUG Time Parameters

The study employed quartile-based statistics and the coefficient of variation (CV) as key measures of variability to capture the range and distribution of test parameters. Including interquartile range (IQR) and CV provided additional robustness by highlighting variability and proportional consistency in the data.

Table 3 presents the total time to complete the iTUG test, which averaged 13.1 ± 1.9 s with a CV of 14.8%, indicating low variability. The standing phase had a mean duration of 1.1 ± 0.3 s and a CV of 29.2%, reflecting average variability. The first walking phase (Walk 1) recorded a mean time of 4.1 ± 1.0 s with a CV of 22.4%, also indicating average variability. Turn 1, a critical phase for balance assessment, had a mean duration of 2.0 ± 0.6 s and a CV of 19.5%, showing low variability. The second walking phase (Walk 2) had a mean time of 3.2 ± 0.7 s with a CV of 20.9%, indicating average variability. Turn 2 phase exhibited a mean duration of 1.1 ± 0.4 s and a CV of 24.1%, also reflecting average variability. The sit phase, marking the final component of the test, averaged 1.6 ± 0.5 s with a CV of 23.2%, indicating average variability. Stand duration (time standing still between movements) had a mean of 1.1 ± 0.2 s and a CV of 20.6%, marking the threshold between low and average variability. Stand flexion duration and stand extension duration, measuring partial body movements, recorded mean times of 0.6 ± 0.2 s (CV of 31.3%) and 0.5 ± 0.2 s (CV of 39.1%), respectively, both indicating average variability. The coefficient of variation (CV) for iTUG test variables revealed diverse levels of variability. Low variability was observed in the total time and Turn 1 phase, indicating consistent performance across participants. Average variability (20% < CV < 40%) was found in the Walk 1 phase, Walk 2 phase, Sit phase, Turn 2 phase, and Stand phase, reflecting moderate inter-individual differences. Notably, high variability (40% < CV < 100%) was seen in Stand Ext. Duration (39.1%) and Stand Flex. Duration (31.3%), suggesting these phases may be influenced by individual motor strategies.

### 3.2. Parameters of Walk 1 and Walk 2 Phases

Table 4 details the recorded temporal and spatial gait variables, alongside kinematic variables of the iTUG test for the test group. The average walk duration was 7.3 ± 1.4 s with a CV of 19.8%, indicating low variability. The mean cadence was 107.6 ± 8.3 steps/min with a CV of 7.7%, exhibiting low variability. The average step count was 11.3 ± 2.3 steps with a CV of 20.2%, indicating average variability. The mean stride time was 1.1 ± 0.1 s, showing low variability (CV of 7.7%). The average stance phase duration on the left was 60.3 ± 3.0% of the gait cycle (CV of 3.3%), indicating low variability. The right stance phase duration averaged 59.6 ± 2.3% (CV of 3.9%), also showing low variability. The left swing phase duration averaged 39.7 ± 2.0% (CV = 5.0%), indicating low variability, while the right swing phase duration averaged 40.5 ± 2.3% (CV of 5.7%), also indicating low variability. The mean single support duration on the left was 39.1 ± 2.3% (CV of 5.7%), indicating low variability. The right single support phase averaged 39.3 ± 3.0% (CV of 7.6%), showing low variability. The first double support phase on the left had a mean duration of 9.7 ± 2.0% (CV of 22.8%), indicating average variability, while the right phase had a mean duration of 10.6 ± 2.4% (CV of 22.8%), also showing average variability. The mean stance ratio (R − L)/(R + L) was −0.7 ± 2.1% with a CV of −313.0%, indicating extremely high variability. The mean double support ratio (R − L)/(R + L) was 4.1 ± 11.0% with a CV of 271.5%, indicating very high variability. The mean pitch angle at left foot contact was −19.1 ± 5.3 degrees, with a CV of 28.1%, indicating average variability. The mean pitch angle at right foot contact was −17.8 ± 4.3 degrees, with a CV of 24.1%, indicating average variability. The mean pitch angle at the left foot off was 56.6 ± 7.8 degrees, with a CV of 14.1%, indicating low variability, while the right foot off mean angle was 55.7 ± 6.8 degrees, with a CV of 12.3%, also showing low variability. The maximum pitch speed for the left foot averaged 40.6 ± 15.6 deg/s, with a CV of 38.4%, indicating average variability. The maximum pitch speed for the right foot averaged 40.6 ± 11.9 deg/s, with a CV of 29.5%, also indicating average variability. The analysis of variance for walking-related variables in the iTUG test revealed consistent results for most temporal and spatial parameters, reflecting low variability in measures such as stride time, stance percentages, and foot pitch speed. Moderate variability was observed in specific support phases, including single and double support durations, indicating some inter-individual differences in gait strategies. High variability emerged in calculated ratios (e.g., stance and double support ratios), suggesting these derived variables are sensitive to asymmetries or outliers. The data highlight that key walking parameters are generally stable, while derived measures may warrant further exploration to interpret variability across individuals.

### 3.3. Turn 1 and Turn 2 Phase Parameters

Table 5 presents the recorded spatio–temporal variables and speed metrics for the Turn 1 and Turn 2 phases of the iTUG test for the test group. For Turn 1, the mean duration was 2.0 ± 0.4 s with a CV of 19.5%, indicating low variability. The average step count was 1.9 ± 0.9 steps with a CV of 45.2%, indicating high variability. The mean stride time was 1.2 ± 0.1 s with a CV of 11.4%, showing low variability. The mean stance phase duration on the left during Turn 1 was 59.1 ± 4.9% of the gait cycle (CV of 8.4%), indicating low variability, while the right stance phase averaged 58.3 ± 6.7% (CV of 11.5%), also showing low variability. The left swing phase duration averaged 40.9 ± 4.9% (CV = 12.2%), indicating low variability, whereas the right swing phase averaged 41.2 ± 4.2% (CV of 10.2%), showing low variability. The left single support phase during Turn 1 averaged 36.7 ± 7.4% (CV of 20.2%), indicating average variability, while the right single support phase averaged 37.3 ± 9.8% (CV of 9.8%), indicating low variability. The first double support phase on the left had a mean duration of 11.5 ± 4.9% (CV of 42.9%), indicating high variability, while the right phase had a mean duration of 10.9 ± 2.9% (CV of 26.9%), indicating average variability. The maximum trunk rotation speed during Turn 1 averaged 67.0 ± 22.8 deg/s, with a CV of 34.0%, indicating average variability. For Turn 2, the mean duration was 1.1 ± 0.1 s, with a CV of 11.1%, indicating low variability. The maximum trunk rotation speed during Turn 2 also averaged 67.0 ± 22.8 deg/s, with a CV of 34.0%, indicating average variability. The CV analysis for spatio–temporal variables and trunk rotation speeds during Turn 1 and Turn 2 in the iTUG test reveals variability across parameters. Turn durations and stance/swing percentages exhibit relatively consistent performance among participants, indicating reliable timing and weight-shifting patterns. In contrast, stride time and single support phases show moderate variability, reflecting individual gait mechanics and motor control strategies. Trunk rotation speeds and step counts demonstrate higher variability, suggesting differences in turning strategies and trunk control.

### 3.4. Parameters of the Sitting Phase (Transition from Standing to Sitting)

Table 6 presents the recorded spatio–temporal variables, trunk range of motion (ROM) in three planes, and trunk movement velocities in the sagittal plane during the iTUG test for the study group. The average sit duration was 1.6 ± 0.4 s with a CV of 23.2%, indicating average variability. The mean duration for sit trunk flexion was 0.6 ± 0.2 s, with a CV of 40.0%, indicating high variability. The sit trunk extension duration averaged 1.0 ± 0.2 s, with a CV of 21.0%, indicating average variability. For trunk ROM, the mean trunk flexion ROM was 51.7 ± 20.1 degrees with a CV of 38.9%, indicating average variability. Trunk lateral flexion ROM averaged 45.9 ± 20.9 degrees, with a CV of 45.6%, indicating high variability. Trunk rotation ROM had a mean of 163.5 ± 70.4 degrees and a CV of 43.1%, reflecting high variability. The average trunk flexion speed was 36.3 ± 44.3 deg/s with a CV of 122.1%, indicating extremely high variability. The maximum trunk flexion speed averaged 152.7 ± 160.1 deg/s with a CV of 104.9%, indicating very high variability. The average trunk extension speed was 8.5 ± 31.7 deg/s with a CV of 373.6%, indicating extremely high variability. The maximum trunk extension speed averaged 92.9 ± 175.9 deg/s, with a CV of 189.4%, indicating extremely high variability. The CV for spatio–temporal variables, trunk range of motion (ROM), and trunk movement velocities during the sitting phase of the iTUG test highlights substantial variability in certain parameters. Sit duration and trunk extension duration exhibit relatively consistent performance, while trunk flexion duration and ROM in all three planes show moderate variability, reflecting inter-individual differences in movement control. Trunk movement velocities, particularly average and maximum speeds, demonstrate significantly higher variability, indicating possible compensatory movements among participants.

## 4. Discussion

The aim of this study was to identify which parameters of the iTUG test, as quantified through motion analysis systems, demonstrate significant variability and distribution patterns relevant to the assessment of musculoskeletal performance in younger adults. By focusing on variability measures, this study provides foundational insights into parameter selection for future diagnostic and normative studies.

Our study highlighted the utility and interpretative value of the iTUG test in clinical practice. The average total time to complete the iTUG test was 13.1 ± 1.9 s with a low coefficient of variation (CV), suggesting consistent performance among participants. This consistency is critical for the iTUG test’s reliability in evaluating overall functional mobility and identifying individuals at risk of falls [26]. Both the stand phase duration and the sit phase duration exhibit average variability. These parameters are essential in assessing lower limb strength and balance during transitional movements, which are often impaired in elderly or neurologically compromised patients. The moderate variability observed in these phases underscores the importance of repeated measures to establish baseline performance and monitor changes over time. The walking phases (Walk 1 and Walk 2) both exhibited average variability. These phases are pivotal for evaluating gait speed and efficiency, which are crucial indicators of mobility and independence in daily activities. The balanced variability in these measures reflects the natural differences in gait patterns among individuals while maintaining a level of consistency that supports their use in clinical settings. Turn 1 and Turn 2 phases are particularly valuable for assessing dynamic balance and postural control. The low variability in Turn 1 suggests a relatively stable performance, which is beneficial for identifying subtle deficits in balance and coordination. The slightly higher variability in Turn 2 may reflect the complexity of the movement, indicating a need for more nuanced analysis in clinical evaluations. Stand duration, along with stand flexion and extension durations, highlight the role of static balance and postural adjustments. The average variability in these parameters indicates a significant degree of inter-individual differences, which could be influenced by various factors such as age, muscular strength, and proprioceptive acuity. These measures may be instrumental in designing targeted interventions aimed at improving static and dynamic balance. What is more, the results highlight that most phases of the iTUG test exhibit low to average variability, with the exception of the stand flexion and extension durations, which approach the higher end of average variability. The low variability in the total time, Turn 1 phase, and stand duration suggests consistent performance across these parameters, reinforcing the iTUG test’s reliability in assessing functional mobility. The high variability in trunk ROM measurements suggests significant differences in movement capabilities among participants, which could be due to varying levels of functional capacity or underlying conditions. In contrast, the average variability in trunk speed metrics indicates more consistent performance in dynamic movements during the iTUG test. These findings highlight the potential of trunk movement analysis for differentiating between levels of functional mobility and for identifying specific impairments.

The temporal variables, such as walk duration and cadence, serve as fundamental indicators of gait efficiency and overall mobility. In clinical settings, deviations from normative data in these parameters can signal underlying issues such as neuromuscular disorders or post-surgical recovery complications. These metrics are particularly useful for identifying patients who may be at risk of falls or who have diminished functional capacity, enabling timely interventions to prevent adverse outcomes. Spatial gait variables, including step count and stride time, further enhance the understanding of gait dynamics. They provide a detailed breakdown of the gait cycle, allowing clinicians to assess symmetry and coordination. For instance, variations in step count and stride time can reveal asymmetries that may not be apparent through simple observation, thus facilitating more precise diagnoses and tailored rehabilitation programmes [32]. Gait stance and swing phases offer insights into balance and stability. The stance phase, representing the portion of the gait cycle where the foot is in contact with the ground, is critical for assessing weight-bearing stability. Meanwhile, the swing phase, where the foot moves forward, reflects the patient’s ability to coordinate limb movement. Abnormalities in these phases can indicate balance impairments or muscle weakness, guiding clinicians in designing specific therapeutic exercises to address these issues. The inclusion of kinematic variables, such as foot pitch at contact and foot off, adds another layer of depth to the assessment. These measurements capture the angles of the foot during key moments of the gait cycle, providing valuable information about joint mobility and muscle function [29]. High variability in these angles can be indicative of compensatory mechanisms due to pain, stiffness, or weakness, offering critical clues for refining treatment plans.

The spatio–temporal and rotational speed parameters of the iTUG test during turns provide comprehensive insights into the Turn 1 and Turn 2 phases of the iTUG test. In clinical practice, the duration and step count during turns are pivotal for evaluating a patient’s dynamic stability and agility [29]. For example, turn duration reflects the time required to execute a change in direction—a task that challenges balance and coordination. An extended turn duration may indicate difficulties in balance or motor control, suggesting a higher fall risk. Similarly, the step count during turns provides additional context; an increased number of steps may signal compensatory strategies employed to maintain balance, revealing potential deficits in stability. Stride time during turns offers insights into the temporal organisation of gait. Inconsistent stride times may denote issues with rhythm and coordination, often observed in neurological disorders such as Parkinson’s. This measure can help clinicians identify deviations from normal gait patterns and tailor interventions accordingly. The stance and swing phases during turns are also critical. The proportion of the gait cycle spent in stance versus swing phases can indicate the efficiency of gait and balance control. A prolonged stance phase or shortened swing phase during turns might suggest a cautious gait pattern, often adopted by individuals with impaired balance to increase stability. Evaluating these phases aids in understanding how patients distribute their weight and manage dynamic balance during direction changes. Single and double support phases during turns are essential for assessing postural stability. Extended single support phases can be a marker of improved balance confidence. In contrast, prolonged double support phases may indicate a need for increased stability, often seen in elderly patients or those with musculoskeletal issues. These phases provide a window into the strategies used by patients to maintain balance, offering clues about their confidence and stability. The rotational speed of the trunk during turns, both average and maximum, is particularly useful for assessing the fluidity and control of rotational movements. Reduced trunk rotation speed can be indicative of rigidity or bradykinesia, commonly seen in patients with Parkinsonian syndromes. Conversely, excessive rotational speed may suggest a lack of control, potentially increasing the risk of imbalance and falls. These metrics often enable clinicians to evaluate the rotational dynamics of gait, which are crucial for tasks requiring changes in direction. In summary, most of the spatio–temporal variables for Turn 1 and Turn 2 exhibit low to average variability, except for the high variability observed in the step count and the first double support phase on the left during Turn 1. The consistent performance in trunk rotation speed across both turns suggests a reliable measurement of rotational dynamics during the iTUG test.

Our results focus on several important aspects regarding the spatio–temporal variables, trunk range of motion (ROM), and trunk movement velocities during the iTUG test. Specifically, the sit duration and trunk movement durations are essential for evaluating the transitional movements between sitting and standing. These phases are particularly relevant in geriatric populations and individuals with mobility impairments, as they often face challenges in these transitions [31]. The observed variability in these parameters underscores the importance of personalised assessments, as patients may exhibit significant differences in their ability to perform these movements smoothly and efficiently. Clinicians can use this information to tailor interventions that focus on improving transitional movements, thereby enhancing overall functional mobility. Trunk ROM measurements in flexion, lateral flexion, and rotation provide valuable insights into the flexibility and dynamic stability of the trunk. High variability in these parameters suggests a wide range of movement capabilities among individuals, which can be indicative of underlying musculoskeletal or neurological conditions. Clinicians can utilise trunk ROM data to identify specific limitations in movement and to develop targeted rehabilitation programmes aimed at improving trunk flexibility and stability. Notably, enhancing trunk ROM is crucial for reducing the risk of falls, as a more flexible and stable trunk can better accommodate balance perturbations [32]. The trunk movement velocities, both average and maximum speeds, are indicative of the dynamic performance of the trunk during the iTUG test. The extremely high variability observed in these parameters highlights the diverse movement strategies employed by individuals. Patients with slower or less-controlled trunk movements may be at a higher risk of falls and may benefit from interventions designed to enhance trunk strength and control. The ability to accurately measure and monitor trunk movement velocities may provide clinicians with a powerful tool for assessing the effectiveness of therapeutic interventions and for making data-driven decisions about patient care. Moreover, in clinical practice, the integration of spatio–temporal variables, trunk ROM, and movement velocities allows for a multidimensional evaluation of mobility, which is essential for identifying specific deficits and tailoring interventions [33]. By focusing on these detailed measurements, clinicians can develop more effective and individualised treatment plans, ultimately improving patient outcomes in terms of mobility, independence, and quality of life.

### Study Limitation

The main limitation is the narrow generalizability of the study. The participants were all young, physically active adults with a BMI under 31. While this is suitable for generating normative data for a specific demographic, it limits the generalizability to other populations, such as older adults, individuals with varying levels of physical activity, or those with higher BMI. Therefore, future studies are needed to establish normative data for other groups, especially those most likely to undergo the iTUG test in clinical settings (e.g., the elderly or patients with musculoskeletal disorders). Although the sample size (N = 73) exceeded the minimum calculated requirement based on the pilot study, it may remain insufficient to fully establish normative values for younger adults. Future research should include larger, multi-centre cohorts to enhance the generalizability of findings. Furthermore, the tests were conducted in a motion analysis laboratory, which may limit the generalizability of the results. The conditions may differ from real-world scenarios where the iTUG is applied, such as clinical settings. A future reliability study is needed to confirm the consistency of the measurements using iTUG [34,35]. Additionally, comparing the results with other established methods (e.g., clinical assessments) could strengthen the study’s conclusions.

## 5. Conclusions

The parameters measured by the iTUG test are invaluable in providing a comprehensive assessment of an FMA. The use of advanced sensor-based technologies, as demonstrated by our iTUG test results, increases the accuracy and reliability of the assessments, making it a potentially important tool in modern clinical practice. Furthermore, the data highlight the robustness and clinical relevance of the iTUG test in different aspects of FMA. The low to moderate variability of most parameters suggests that the iTUG test provides reliable and consistent measurements, making it a valuable tool for both baseline assessment and longitudinal monitoring. The results of this study highlight the potential of the iTUG test as a practical tool in the clinical setting for assessing functional mobility and musculoskeletal performance. The low variability observed in key parameters, such as total time and percentage of posture between subjects in the observation group, suggests that the iTUG test provides reliable, objective, and reproducible measurements in a cross-sectional assessment of healthy young adults. However, as our study focuses on inter-individual variability rather than within-subject reproducibility, further research is required to confirm its test–retest reliability.

## Figures and Tables

**Table 1:** The position of the sensors on the body of the tested individual.

Region	Inertial Unit Positions
Sternum	At the level of the sternum or breastbone
Mid-spine	At the mid-point of the spine, around the level of the thoracic vertebrae
Lower spine	At the lumbar region of the spine
Left and Right shoulder	On each shoulder on the acromion—the bony prominence at the top of the shoulder
Left/Right upper arm	On the lateral side of each upper arm, midway between the shoulder and the elbow
Left/Right forearm	On the lateral side of each forearm, typically halfway between the elbow and the wrist
Left/Right hand	On the dorsum (back) of each hand, near the wrist
Left/Right thigh	On each thigh, midway between the hip and knee, on the lateral side
Left/Right shank	On the lateral side of each shank, midway between the knee and the ankle

**Table 2:** Parameters characterising the Timed Up and Go (TUG) Test, including definitions and interpretations.

Parameter	Unit	Definition	Interpretation and Clinical Significance
*Stand Phase (transition from sitting to standing)*			
Stand Duration	s	Time taken to transition from sitting to standing	Duration indicates the efficiency and control in transitioning to a standing position
Stand Flexion Duration	s	Duration of trunk flexion during the stand	Longer durations may indicate difficulty in initiating or maintaining flexion
Stand Extension Duration	s	Duration of trunk extension during the stand	Duration reflects control over trunk extension during standing
Trunk Flexion ROM	deg	Range of motion of the trunk in flexion	Greater ROM indicates greater flexibility and mobility
Trunk Lateral ROM	deg	Lateral range of motion of the trunk	Greater lateral ROM may reflect trunk stability
Trunk Rotation ROM	deg	Rotational range of motion of the trunk	Greater rotational ROM may reflect rotational mobility
Trunk Flexion Speed Avg	deg/s	Average speed of trunk flexion	Higher speeds suggest greater control and dynamics of trunk flexion
Trunk Flexion Speed Max	deg/s	Maximum speed of trunk flexion	Higher peak speed reflects better control and strength in trunk flexion
Trunk Extension Speed Avg	deg/s	Average speed of trunk extension	Higher speeds suggest greater control and dynamics of trunk extension
Trunk Extension Speed Max	deg/s	Maximum speed of trunk extension	Higher peak speed reflects better control and strength in trunk extension
*Walk 1 and Walk 2 Phases*			
Walk Duration	s	Time taken to walk the 3-metre distance	A shorter duration indicates faster and potentially more efficient walking
Walk Cadence	steps/min	Number of steps per minute	Higher cadence reflects a faster pace
Walk Step Count	steps	Total number of steps taken	A lower step count may indicate efficient gait with adequate stride length, stability, and confidence in walking ability
Walk Stride Time	s	Time taken for one complete gait cycle	Shorter stride times indicate quicker gait cycles
Walk Stance	%	Percentage of the gait cycle spent in stance phase, recorded separately for left and right legs	Reduced stance phase percentage can reflect a quicker gait or increased confidence in single-leg support
Walk Swing	%	Percentage of the gait cycle spent in swing phase, recorded separately for left and right legs	Higher swing phase percentage may indicate a faster gait with shorter stance duration
Walk Single Support	%	Percentage of the gait cycle spent on single-leg support, recorded separately for left and right legs	Higher percentage reflects balance in single-leg stance
Walk 1st Double Support	%	Percentage of the gait cycle spent on double-leg support	Higher double support indicates stability during the weight acceptance phase
Walk Foot Pitch at Contact	deg	Angle of the foot at initial ground contact, recorded separately for left and right feet	Foot angle reflects gait and balance during contact
Walk Stance Ratio	%	Ratio of stance times between the right and left legs	Closer to 100% indicates equal stance times; a measure of asymmetry
Walk Double Support Ratio	%	The ratio of double support times between the right and left legs	Closer to 100% indicates equal double support time, a measure of asymmetry
Walk Foot Pitch at Foot Off	deg	Angle of the foot at the moment it leaves the ground, recorded separately for left and right feet	Reflects foot dynamics at foot-off or efficiency of push-off
Walk Foot Pitch Speed Max	deg/s	Maximum speed of foot pitch during walking, recorded separately for left and right feet	Higher values indicate greater control in foot movement
*Turn 1 and Turn 2 Phases*			
Turn 1 Duration	s	Time taken to complete the first turn	A shorter time indicates efficient turning
Turn 1 Step Count	steps	Number of steps taken during the first turn	A higher count indicates more steps needed for turning
Turn 1 Stride Time	s	Time taken for one stride during the turn	Shorter stride time indicates quicker movement
Turn 1 Stance	%	Percentage of the turn spent in stance phase, recorded separately for left and right legs	A higher stance percentage suggests stability during the turn
Turn 1 Swing	%	Percentage of the turn spent in swing phase, recorded separately for left and right legs	A higher swing percentage indicates a more dynamic turn
Turn 1 Single Support	%	Percentage of the turn spent on single-leg support, recorded separately for left and right legs	A higher support percentage indicates balance on one leg
Turn 1 1st Double Support	%	Percentage of the turn spent on double-leg support	Higher values indicate balanced turning support
Turn 1 Trunk Rotation Speed Max	deg/s	Maximum speed of trunk rotation during the first turn	Higher values suggest rapid trunk rotation control
Turn 2 Duration	s	Time taken to complete the second turn	A shorter duration indicates an efficient final turn
Turn 2 Trunk Rotation Speed Max	deg/s	Maximum speed of trunk rotation during the second turn	Higher speed suggests agility in trunk rotation
*Sit Phase (transition from standing to sitting)*			
Sit Duration	s	Time taken to transition from standing to sitting	A shorter time suggests control and efficiency in sitting
Sit Trunk Flexion Duration	s	Duration of trunk flexion during the sit	Longer flexion duration may indicate difficulty or cautious movement
Sit Trunk Extension Duration	s	Duration of trunk extension during the sit	Reflects control during trunk extension in sitting
Sit Trunk Flexion ROM	deg	Range of motion of the trunk in flexion during the sit	Greater ROM indicates flexibility and mobility
Sit Trunk Lateral ROM	deg	Lateral range of motion of the trunk during the sit	Higher lateral ROM may reflect trunk stability
Sit Trunk Rotation ROM	deg	Rotational range of motion of the trunk during the sit	Reflects rotational mobility
Sit Trunk Flexion Speed Avg	deg/s	Average speed of trunk flexion during the sit	Higher speeds suggest better trunk control
Sit Trunk Flexion Speed Max	deg/s	Maximum speed of trunk flexion during the sit	Higher peak speed reflects agility in trunk flexion
Sit Trunk Extension Speed Avg	deg/s	Average speed of trunk extension during the sit	Higher speeds suggest controlled extension
Sit Trunk Extension Speed Max	deg/s	Maximum speed of trunk extension during the sit	Higher peak speed reflects controlled trunk extension during sitting

**Table 3:** Recorded results of time parameters for the instrumented Time Up and Go measurement for the test group and for the phases of sit-to-stand, walking, turn 1 and turn 2, and stand-to-sit.

Variable	Mean	Median	SD	Minimum	Maximum	Lower Quartile	Upper Quartile	Quartile Range	Coef.Var.
Total time [s]	13.1	12.7	1.9	9.9	17.7	11.6	14.6	3.0	14.8
Stand phase [s]	1.1	1.1	0.3	0.6	2.9	0.9	1.2	0.3	29.2
Walk 1 phase [s]	4.1	4.2	0.9	2.4	6.7	3.4	4.7	1.4	22.4
Turn 1 phase [s]	2.0	2.0	0.4	1.0	3.1	1.7	2.2	0.6	19.5
Walk 2 phase [s]	3.2	3.1	0.7	1.7	5.0	2.7	3.7	1.0	20.5
Turn 2 phase [s]	1.1	1.1	0.3	0.7	2.2	0.9	1.3	0.4	24.1
Sit phase [s]	1.6	1.5	0.4	0.9	2.7	1.3	1.8	0.5	23.2
Stand Duration [s]	1.1	1.1	0.3	0.6	2.9	0.9	1.2	0.3	29.2
Stand Flex. Duration [s]	0.6	0.6	0.2	0.2	1.7	0.5	0.7	0.2	31.3
Stand Ext. Duration [s]	0.5	0.4	0.2	0.2	1.2	0.4	0.6	0.2	39.1

Abbreviations Coef.Var. = coefficient of variation (Std.Dev/Mean) in percentage. Interpretation of Coef.Var: CV < 20%—low variability; 20% < CV < 40%—average variability; 40% < CV < 100%—high variability; 100% < CV < 150%—very high variability; CV > 150%—extremely high variability.

**Table 4:** Recorded results of time–space and gait phase variables and selected angular parameters of Walk 1 and Walk 2 phases.

Variable	Mean	Median	SD	Minimum	Maximum	Lower Quartile	Upper Quartile	Quartile Range	Coef.Var.
Walk Duration [s]	7.3	7.3	1.4	4.8	10.6	6.0	8.5	2.5	19.8
Walk Cadence [steps/min]	107.6	108.1	8.3	91.3	122.4	101.3	113.9	12.5	7.7
Walk Step Count [steps]	11.3	11.0	2.3	8.0	16.0	10.0	13.0	3.0	20.2
Walk Stride Time [s]	1.1	1.1	0.1	1.0	1.3	1.0	1.2	0.1	7.7
Walk Stance left [%]	60.3	60.4	2.0	55.3	64.9	58.9	61.9	3.0	3.3
Walk Stance right [%]	59.6	59.6	2.3	52.2	63.6	58.1	61.2	3.1	3.9
Walk Swing left [%]	39.7	39.6	2.0	35.1	44.7	38.1	41.1	3.0	5.0
Walk Swing right [%]	40.5	40.4	2.3	36.4	47.8	38.8	41.9	3.1	5.7
Walk Single Support left [%]	39.1	40.7	8.6	0.0	48.0	38.4	42.7	4.3	22.1
Walk Single Support right [%]	39.3	39.2	3.0	27.5	45.8	38.0	41.1	3.1	7.6
Walk 1st Double Support left [%]	9.7	9.5	2.0	4.8	14.2	8.7	11.0	2.4	20.3
Walk 1st Double Support right [%]	10.6	10.5	2.4	5.7	19.5	9.0	12.0	3.0	22.8
Walk Stance Ratio (R − L)/(R + L) [%]	−0.7	−0.5	2.1	−10.9	3.1	−1.7	0.6	2.3	−313.0
Walk Double Support Ratio (R − L)/(R + L) [%]	4.1	3.3	11.0	−23.6	46.8	−2.5	9.6	12.1	271.5
Walk Foot Pitch At Contact left [deg]	−19.1	−17.3	5.3	−35.6	−11.2	−21.4	−15.2	6.3	−28.0
Walk Foot Pitch At Contact right [deg]	−17.8	−18.1	4.3	−26.8	−10.5	−21.1	−14.2	6.9	−24.3
Walk Foot Pitch At Foot Off left [deg]	56.6	56.7	8.0	34.6	71.7	51.6	62.7	11.1	14.1
Walk Foot Pitch At Foot Off right [deg]	55.4	55.2	7.5	35.8	73.3	50.6	59.2	8.6	13.6
Walk Foot Pitch Speed Max left [deg]	40.2	39.6	6.4	22.8	52.0	35.9	45.7	9.8	15.8
Walk Foot Pitch Speed Max right [deg]	40.6	40.5	5.3	29.5	50.6	36.8	44.7	7.9	13.1

Abbreviations: Coef.Var. = coefficient of variation (Std.Dev/Mean) in percentage. Interpretation of Coef.Var: CV < 20%—low variability; 20% < CV < 40%—average variability; 40% < CV < 100%—high variability; 100% < CV < 150%—very high variability; CV > 150%—extremely high variability.

**Table 5:** Recorded results of the spatio–temporal variables and speed of turn 1 and turn 2 of the instrumented Time Up and Go test for the test group.

Variable	Mean	Median	SD	Minimum	Maximum	Lower Quartile	Upper Quartile	Quartile Range	Coef.Var.
Turn 1 Duration [s]	2.0	2.0	0.4	1.0	3.1	1.7	2.2	0.6	19.5
Turn 1 Step Count [steps]	1.9	2.0	0.9	0.0	5.0	1.0	2.0	1.0	45.2
Turn 1 Stride Time [s]	1.2	1.2	0.1	0.7	1.4	1.1	1.3	0.2	11.4
Turn 1 Stance left [%]	59.1	59.1	5.0	47.5	68.6	56.0	62.4	6.4	8.4
Turn 1 Stance right [%]	58.8	58.3	4.2	50.5	67.9	55.8	61.7	5.9	7.1
Turn 1 Swing left [%]	40.9	40.9	5.0	31.4	52.5	37.6	44.0	6.4	12.2
Turn 1 Swing right [%]	41.2	41.7	4.2	32.1	49.5	38.3	44.2	5.9	10.2
Turn 1 Single Support left [%]	36.7	37.9	7.4	0.0	44.9	34.0	40.5	6.6	20.2
Turn 1 Single Support right [%]	37.5	37.3	3.7	31.3	46.9	34.9	39.5	4.5	9.8
Turn 1 1st Double Support left [%]	11.5	11.2	4.9	4.5	36.0	9.4	12.3	2.9	42.9
Turn 1 1st Double Support right [%]	10.9	11.0	2.9	5.1	15.5	8.5	13.4	4.9	26.9
Turn 1 Trunk Rotation Speed Max [deg/s]	67.0	66.0	22.8	31.9	144.7	50.4	77.3	26.9	34.0
Turn 2 Duration [s]	1.1	1.1	0.3	0.7	2.2	0.9	1.3	0.4	24.1
Turn 2 Trunk Rotation Speed Max [deg/s]	67.0	66.0	22.8	31.9	144.7	50.4	77.3	26.9	34.0

Abbreviations: Coef.Var. = coefficient of variation (Std.Dev/Mean) in percentage. Interpretation of Coef.Var: CV < 20%—low variability; 20% < CV < 40%—average variability; 40% < CV < 100%—high variability; 100% < CV < 150%—very high variability; CV > 150%—extremely high variability.

**Table 6:** Recorded results of spatio–temporal variables, trunk range of motion in three planes, and trunk movement velocities in the sagittal plane of the instrumented Time Up and Go test for the study group.

Variable	Mean	Median	SD	Minimum	Maximum	Lower Quartile	Upper Quartile	Quartile Range	Coef.Var.
Sit Duration [s]	1.6	1.5	0.4	0.9	2.7	1.3	1.8	0.5	23.2
Sit Trunk Flex. Duration [s]	0.6	0.6	0.2	0.2	1.2	0.4	0.7	0.3	40.0
Sit Trunk Ext. Duration [s]	1.0	1.0	0.2	0.5	1.6	0.9	1.1	0.2	21.0
Sit Trunk Flex. ROM [deg]	51.7	48.5	20.1	20.9	132.5	37.1	64.2	27.1	38.9
Sit Trunk Lat. ROM [deg]	45.9	43.2	20.9	15.1	113.6	29.8	55.4	25.6	45.6
Sit Trunk Rot. ROM [deg]	163.5	151.2	70.4	30.5	359.7	118.3	183.4	65.2	43.1
Sit Trunk Flex. Speed Avg [deg/s]	36.3	41.6	44.3	−71.4	151.0	16.8	65.1	48.3	122.1
Sit Trunk Flex. Speed Max [deg/s]	152.7	111.2	160.1	−27.6	1101.6	79.2	166.9	87.7	104.9
Sit Trunk Ext. Speed Avg [deg/s]	8.5	19.3	31.7	−58.7	54.0	−13.0	33.6	46.6	373.6
Sit Trunk Ext. Speed Max [deg/s]	92.9	66.1	175.9	10.2	1520.0	53.4	81.2	27.8	189.4

Abbreviations: Coef.Var. = coefficient of variation (Std.Dev/Mean) in percentage. Interpretation of Coef.Var: CV < 20%—low variability; 20% < CV < 40%—average variability; 40% < CV < 100%—high variability; 100% < CV < 150%—very high variability; CV > 150%—extremely high variability.

## Data Availability

The data presented in this study are available on request from the corresponding author.
